# Elevated Level of Circulating but Not Urine S100A8/A9 Identifies Poor COVID-19 Outcomes

**DOI:** 10.1021/acsinfecdis.3c00249

**Published:** 2023-10-03

**Authors:** Leah Mellett, Gaya Amarasinghe, Christopher W. Farnsworth, Shabaana A. Khader

**Affiliations:** † Department of Molecular Microbiology, Washington University in St. Louis, St. Louis, Missouri 63108, United States; ‡ Department of Pathology and Immunology, Washington University School of Medicine, St. Louis, Missouri 63108, United States; § Department of Microbiology, University of Chicago, Chicago, Illinois 60637 United States

**Keywords:** S100A8/A9, calprotectin, COVID-19, prognosis, biomarker

## Abstract

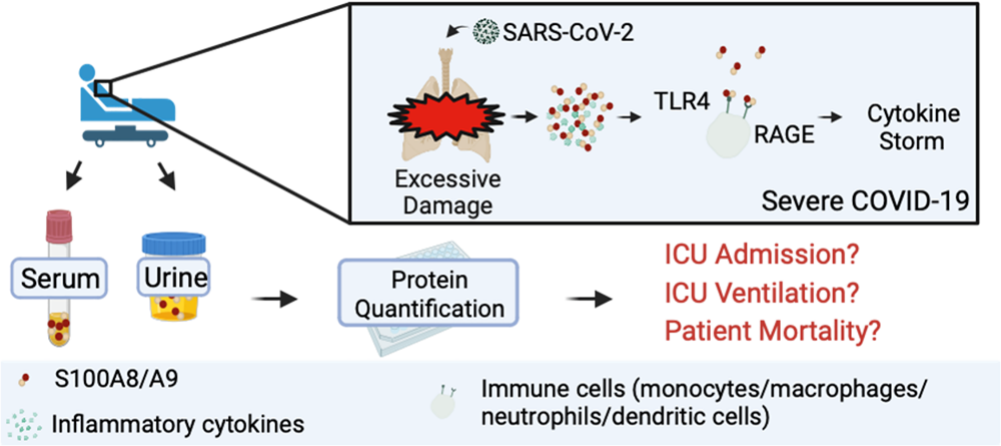

The alarmin calprotectin (S100A8/A9) is thought to drive a cytokine storm, a hallmark of severe COVID-19. Recent studies report circulating S100A8/A9 levels can distinguish COVID-19 severity but have only been conducted in non-U.S. cohorts and mainly focus on serum S100A8/A9 levels. Thus, we quantified S100A8/A9 in serum and urine samples from a hospital cohort in St. Louis, Missouri, to expand the understanding of S100A8/A9 as a prognostic biomarker for COVID-19. Elevated S100A8/A9 serum levels were observed in ICU patients (*n* = 49, *p* = 0.0370) and patients with fatal cases of COVID-19 (*n* = 76, *p* = 0.0018). We observed no correlation in the S100A8/A9 levels in matched serum and urine samples. Our results support the association of serum S100A8/A9 levels with COVID-19 severity and suggest that further investigation of urine S100A8/A9 as a COVID-19 biomarker is not warranted.

Coronavirus disease 2019 (COVID-19) presents heterogeneously in patients, emphasizing an unmet need for specific prognostic tools to accurately assess patient outcomes and disease severity.^1−3^ Identifying the drivers that influence negative patient prognoses is essential for developing effective treatment strategies to improve patient outcomes. A hallmark of severe and fatal COVID-19 cases includes cytokine storm, in which the release of over 150 inflammatory cytokines results in the overactivation and hyperproliferation of macrophages, T-cells, and NK cells.^4,5^ Clinically, cytokine storm presents as the sudden worsening of symptoms due to dysregulated immune response, and can result in sepsis, multiple organ failure, and death.^6−8^ Because cytokine storm is typically amplified by neutrophils, S100A8/A9 has been investigated and associated with poor COVID-19 patient outcomes.^9−12^

S100A8/A9 is an alarmin that accounts for ∼45% of cytoplasmic proteins in human neutrophils.^13,14^ Secretion of S100A8/A9 can occur following inflammation due to metabolic inflammatory conditions, degenerative and autoimmune diseases, and infection.^15^ S100A8/A9 binds to toll-like receptor 4 and the receptor for advanced glycation end products upstream of TNFα and CXCL8 secretion and promotes NF-ΚB signaling, the secretion of inflammatory proteins, and the modulation of leukocyte chemotaxis.^13,16^ In the context of COVID-19, S100A8/A9 has been suggested to initiate cytokine storm by driving IL-6 secretion.^17−22^

While recent studies report the association of circulating S100A8/A9 levels with poor COVID-19 patient outcomes and demonstrate its ability to differentiate COVID-19 severity, these studies have only been conducted in non-U.S. patient cohorts and specifically analyzed samples collected from patients at the time of admission to emergency departments.^2,9,10,12,23,24^ We have previously observed 2-fold variations in serum S100A8/A9 levels in active tuberculosis patients in different geographical cohorts.^25,26^ Therefore, the reported predictive capacities and threshold values of serum S100A8/A9 for the prognostication of COVID-19 patients from non-U.S. cohorts cannot be blindly applied to U.S. cohorts. Furthermore, investigations limited their exploration to the prognostic value of circulating S100A8/A9 levels and did not evaluate the utility of these levels in urine samples.^2,9,10,12,23,24,27−34^

Because S100A8/A9 is involved in multiple inflammatory conditions, such as lupus and diabetes, it has been detected and quantified in urine samples.^35−38^ Still, it is unclear whether urine S100A8/A9 levels are indicative of COVID-19 severity or patient prognosis. Therefore, we quantified S100A8/A9 levels from hospitalized Saint Louis COVID-19 patients to validate previous findings from non-U.S. cohorts and explore a potential non-invasive prognostic biomarker.

First, we obtained banked peripheral blood samples from a cohort of 73 hospitalized patients who were positive by RT-PCR for SARS-CoV-2 (COVID-19 patients) (Table S1) and remnant samples from healthy controls (HCs, *n* = 20; Table S2). Serum S100A8/A9 levels were quantified as previously described and compared with levels in HC samples.^26^ The averages of serum S100A8/A9 concentrations in COVID-19 patients (15580.7 ng/mL) were 9-fold higher compared to HCs (1662.6 ng/mL) (Figure 1A). Samples included in the COVID-19 cohort were collected at different times during a patient’s hospitalization. To determine if length of hospitalization impacted S100A8/A9 levels, we stratified samples by their collection period and found that S100A8/A9 serum levels were significantly different across different lengths of hospitalization (*p* = 0.0065) (Figure 1B). Average serum S100A8/A9 levels in samples taken at 0 days (15580.7 ng/mL), 3 days (16087.7 ng/mL), 7 days (15946.7 ng/mL), and 14 days (14714.9 ng/mL) after hospital admission were significantly elevated in comparison to samples obtained 90+ days post-admission (3691.5 ng/mL), in contrast to samples collected at 28 days post-admission (13522.4 ng/mL) (Figure 1B).

**Figure 1:**
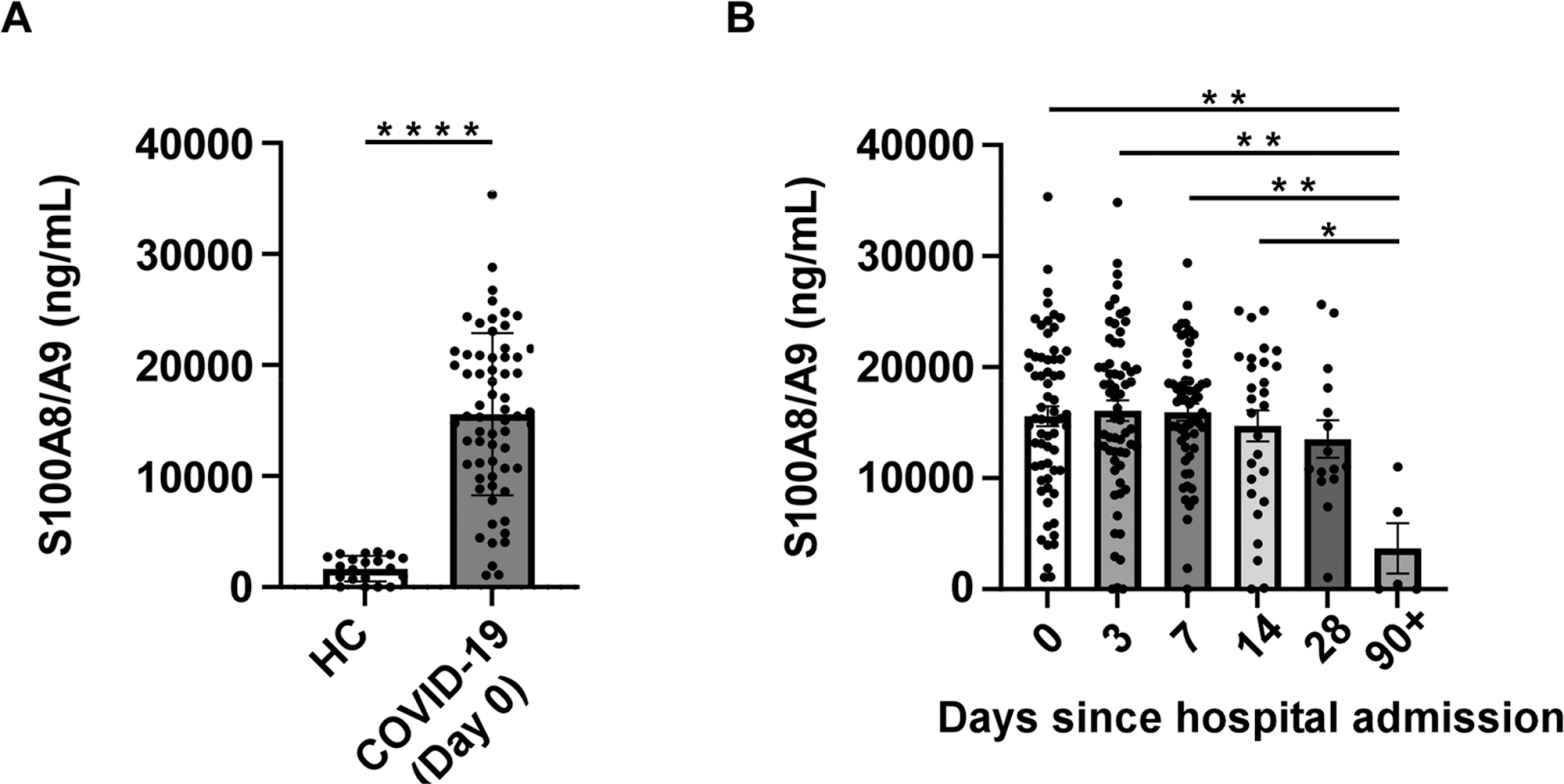
(A) S100A8/A9 serum levels are significantly elevated in COVID-19 Day 0 serum samples compared to HCs; Mann–Whitney U test. Day 0 is the time of a patient’s hospital admission. (B) S100A8/A9 serum levels decrease in COVID-19 patients after 90+ days of hospitalization; ordinary one-way ANOVA with Tukey’s multiple comparisons. Data shown are the mean ± SEM. *P ≤ 0.05; **P ≤ 0.01; ****P ≤ 0.0001.

To analyze the effects of the length of hospitalization and patient mortality status, ICU admission, or ICU ventilation on serum S100A8/A9 levels, we performed two-way ANOVA analyses that revealed there were no statistically significant interactions between these variables (Figure 2, Table S3). Consistent with published findings, simple main effects analyses showed that S100A8/A9 levels were significantly elevated in patients that required ICU admission (*p* = 0.0370) and those with fatal COVID-19 cases (*p* = 0.0018) (Figure 2A,B, Table S3). In contrast, there was no significant difference in S100A8/A9 serum levels between ICU patients that required ventilation compared to those who did not (*p* = 0.0647) (Figure 2C). The average S100A8/A9 serum levels in deceased COVID-19 patients, patients admitted to the ICU, and patients that required ICU ventilation were higher compared to patients with favorable disease course (Figure 2 insets). Additionally, serum S100A8/A9 levels were significantly increased in patients with higher BMIs, which were recorded at the time of hospital admission (*p* = 0.0338, Table S4). Notably, we found that serum S100A8/A9 levels were significantly related to mortality status and ICU admission, independent of hospitalization length (Figure 2, Table S3) and patient BMI (Table S5).

**Figure 2:**
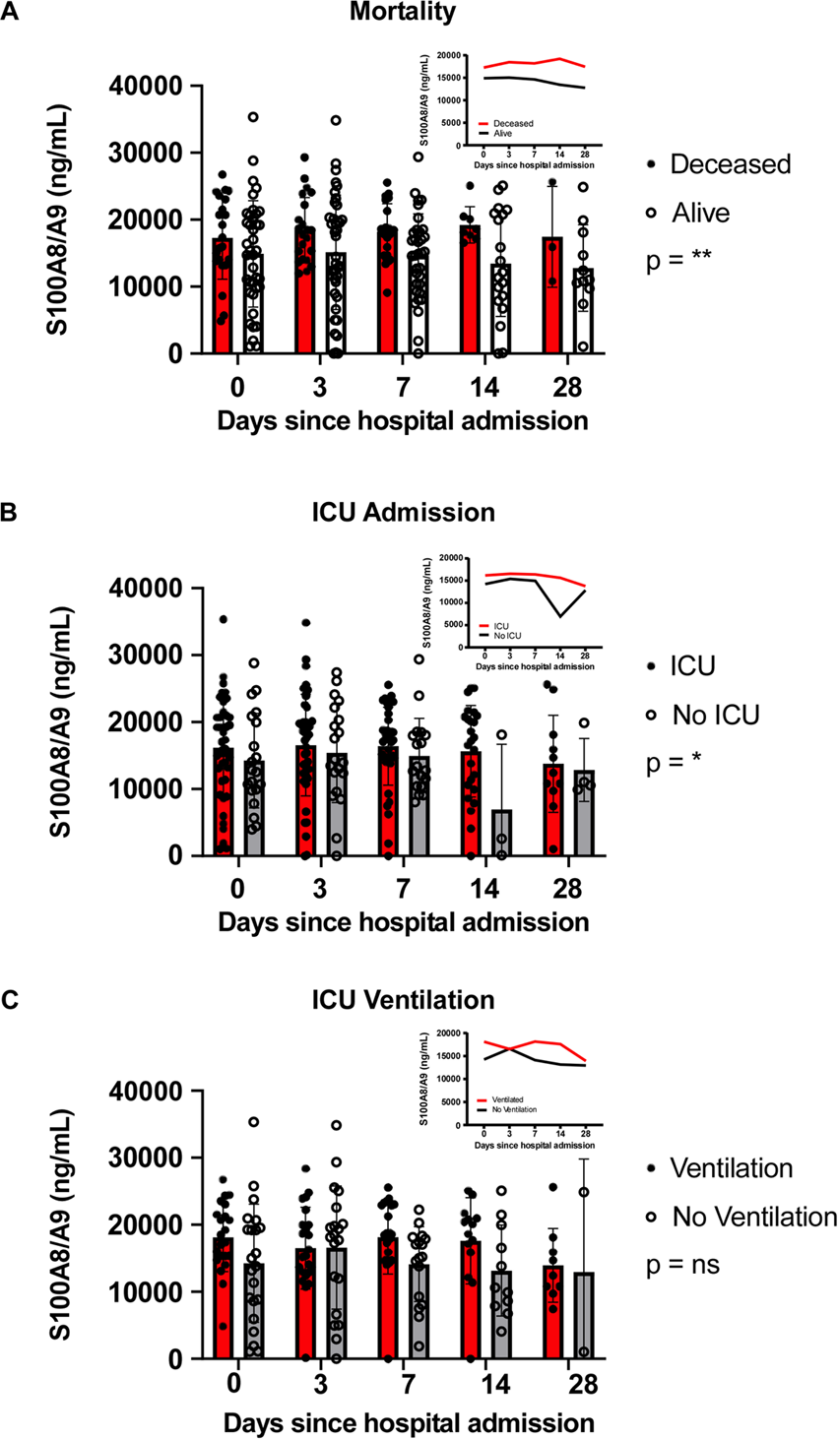
S100A8/A9 quantification of COVID-19 serum samples stratified by (A) patient mortality outcome, (B) patient ICU admission status, and (C) patient ICU ventilation status. Insets: average S100A8/A9 values across the hospitalization period. All studied via two-way ANOVA with Šídák’s multiple comparison. Data shown are mean ± SEM. Listed *p*-values represent simple main effects. *P ≤ 0.05; **P ≤ 0.01; ****P ≤ 0.0001; ns = not significant.

In contrast to published studies, we did not observe significant serum S100A8/A9 variation related to length of hospitalization or ventilation requirement.^11,24,39^ However, this lack of variation may be advantageous, as routine biomarkers such as CRP and procalcitonin may be influenced by potential concomitant bacterial co-infection.^24^ These conclusions are supported by previous studies that observed elevated S100A8/A9 levels in severe COVID-19 patients occurring irrespective of concomitant bacterial co-infections.^9^

Elevated S100A8/A9 urine and serum levels have been observed in patients with active lupus nephritis, and published studies have observed correlations between serum and urine S100A8/A9 levels in patients with Type II diabetes, suggesting that these levels may be related across different conditions.^37,40^ Therefore, we determined if urine S100A8/A9 was correlated to serum S100A8/A9 and indicative of patient outcomes by analyzing matched urine samples from 45 of the 73 COVID-19 patients (*n* = 84). We observed no significant relationship between urine and serum S100A8 and A9 levels across all paired samples (Figure 3). These findings persisted when samples were stratified further by length of hospitalization (Figure S1). Urine S100A8/A9 levels were unable to distinguish between patients when stratified by length of hospitalization and mortality outcome, ICU admission status, or ventilation status (Table S6).

**Figure 3:**
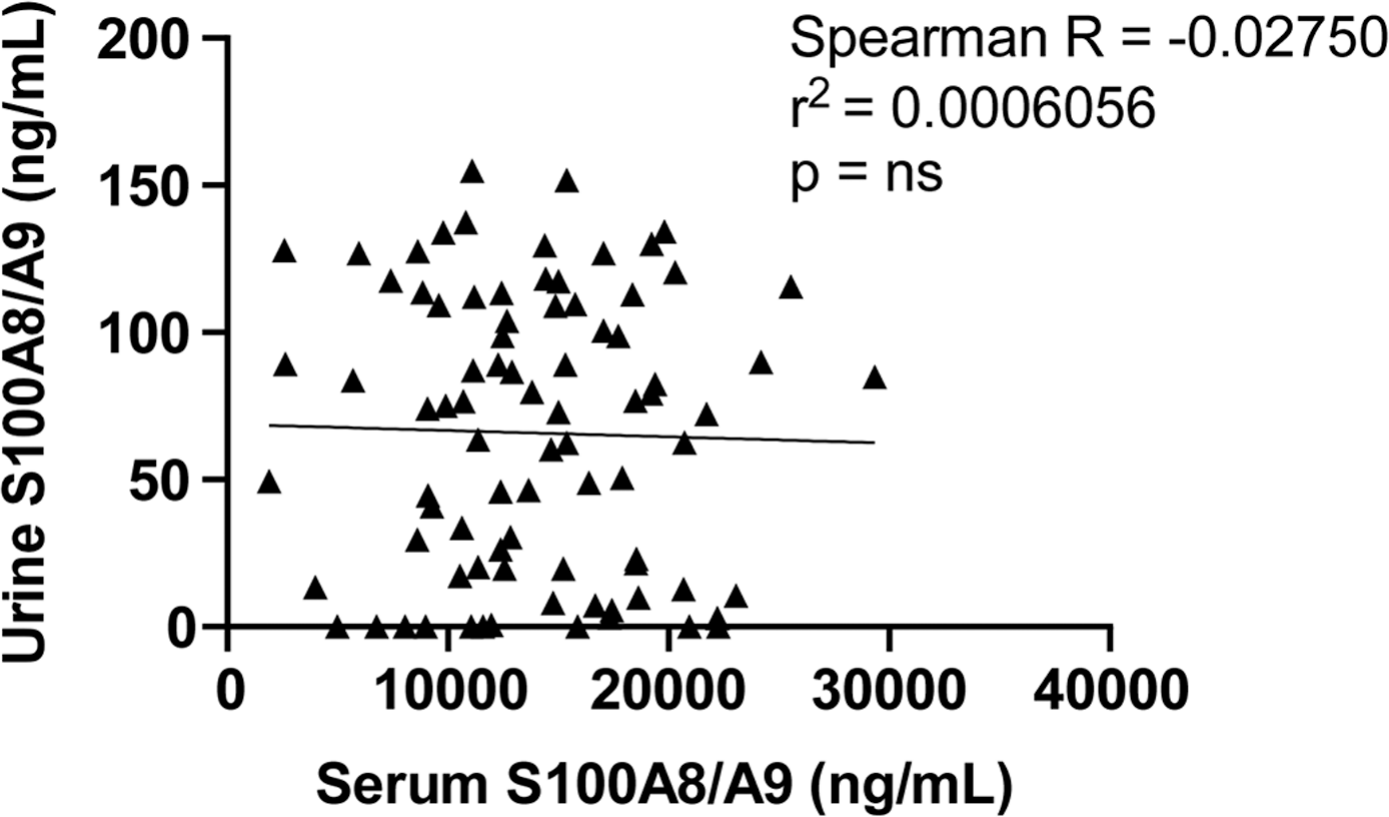
Correlation S100A8 and S100A9 quantification analyses of matched urine and serum samples from COVID-19 patients. Non-parametric simple correlation and linear regression analysis. ns = not significant.

Non-invasive biomarkers allow for the evaluation of patients in various socioeconomic environments and increased patient comfort, and recent studies have focused on investigating urine, as its composition is mainly ultrafiltrate and waste products from the blood that often reflect systemic changes. Our study is the first to demonstrate that while serum S100A8/A9 levels are elevated during severe COVID-19, these levels are not correlated in matched urine samples. Importantly, our findings are in contrast to previous studies that found a correlation between serum and urine S100A8/A9 levels in diabetic patients.^37^ This discrepancy indicates that the association of S100A8/A9 in urine and serum samples may be disease-dependent rather than a universally observed phenomenon. Future studies should consider normalizing to creatinine levels to better delineate the utility of urine S100A8/A9.

Limitations of our study include the lack of substantial age overlap between COVID-19 and healthy cohorts and the lack of normalization to urine creatinine levels. It has been suggested that S100A8/A9 is implicated in age-related inflammation, and future studies should consider this demographic in their prospective investigations. Additionally, our study did not include a positive control to validate the use of our assay or the sample dilution used for urine S100A8/A9 quantification. Future investigations should include these samples to support their findings and allow for comparisons across different indications.

Overall, our study provides a deeper understanding of how peripheral S100A8/A9 levels relate to COVID-19 patient outcomes in the U.S. and offers insight into the association between urine and serum S100A8/A9 levels. Our results help direct future diagnostic and prognostic investigations of S100A8/A9 during COVID-19 and improve the field’s understanding regarding the utility of urine S100A8/A9 as a potential biomarker, especially in diseases that currently use serum S100A8/A9 as a marker of patient outcomes.

## Methods

### COVID-19 Patient Cohort

Banked frozen samples were obtained from the WU350 COVID-19 study and distributed through the Tissue Procurement Center (TPC) and the Henderson Lab at Washington University (WU) in Saint Louis. The WU350 study collected samples from 350 patients at Barnes Jewish Hospital (BJH) who presented with respiratory illness symptoms and returned a positive physician-ordered SARS-CoV-2 RNA PCR test between March 26 and August 28, 2020. Samples and clinical data from COVID-19 patients were collected from consenting hospitalized patients at the WU Medical Center under an IRB-approved protocol (ID# 202003085) to study immunological, genetic, and clinical predictors of SARS-CoV-2 infection with assistance from the WU Institute of Clinical and Translational Sciences.

To define populations of interest within the WU350 study, five patient groups were created based on the following inclusion criteria: Group 1: symptomatic participants, 18 years or older, who present to BJH emergency departments (or affiliated testing site) AND for whom physician-initiated SARS-CoV-2 testing is requested; Group 2: symptomatic participants, 18 years or older, who present to BJH emergency departments and test positive for seasonal coronavirus on respiratory viral panel testing; Group 3: household contacts, 18 years or older, of enrolled symptomatic participants who test positive for SARS-Co-V-2; Group 4: pediatric patients (12 months–18 years), who present to St. Louis Children’s Hospital and test positive for SARS-CoV-2; Group 5: residents of nursing homes/skilled nursing facilities/long-term acute care facilities. Additional inclusion criteria: the ability to complete study procedures and the ability to understand and give informed consent (or have consent provided by a legal authorized representative) or parent/guardian for children under 18 years. Patients who were incarcerated, unable to provide consent/do not have an appropriate surrogate, received immunoglobulin or other blood products within 90 days prior to study enrollment (with the exception of Rho D immunoglobulin), donated blood/blood products within 30 days prior to study enrollment, had a condition that may interfere with the proper conduct of the trial (based on investigator opinion), or enrolled in foster care/were Wards of the State were excluded.

Samples obtained from the TPC and used in this study (*n* = 233) were collected under an IRB-approved protocol (ID# 202103117) from 73 patients, and selection of these samples was prioritized for analysis based on the availability of matched urine samples. Information pertaining to WU350 patient subpopulation groups 1–5 was not provided to us for this study, nor was it a factor in sample selection. Patients with diabetes or lupus were excluded from our study based on previous publications that reported elevated S100A8/A9 urine levels in these populations.^35−38^ Patient demographics from the samples utilized in our study are listed in Table S1.

### Serum Sample Collection and Processing

Under the initial WU350 study, serum samples (SST tubes) from COVID-19 patients were collected between the hours of 5 a.m. and 2:30 p.m. and were delivered to the TPC. Samples were stored at room temperature until arrival at the TPC for processing. All samples were processed according to SOP-201 (centrifugation at 1300*g* for 5 min at room temperature) within 6–8 h of collection and aliquoted into 1.5 mL tubes for storage at −80 °C. Specimens were distributed in 200 μL aliquots (2 freeze/thaw cycles) before analysis. Samples were delivered to the TPC at one of two delivery times (10 a.m. or 2 p.m.). Samples that arrived at 10 a.m. were collected between 2 p.m. of the previous day and 10 a.m. the day they were delivered to the TPC. Samples that arrived at 2 p.m. were collected between 10 a.m. and 2 p.m. the day they were delivered to the TPC. De-identified clinical data were provided from electronic health records by the Institute of Informatics.

Healthy serum samples were collected at BJH or affiliated outpatient centers from outpatients with normal estimated glomerular filtration rates/serum creatinine concentrations and no documentation of infection at the time of collection (*n* = 20). These remnant, banked samples were obtained from the Barnes Jewish Hospital Core laboratory, aliquoted into separate tubes, and stored at −80 °C until analysis.

### Urine Sample Collection and Processing

Under the initial WU350 study, patient urine samples were collected in a sterile cup by the hospital staff. Samples were delivered to the TPC at one of two delivery times (10:30 a.m. or 2:30 p.m.). Samples that arrived at 10:30 a.m. were collected between 3 p.m. the previous day and 10:30 a.m. the day they were delivered to the TPC. Samples that arrived at 2:30 p.m. were collected between 10:30 a.m. and 2:30 p.m. the day they were delivered to the TPC. Samples were then transferred to Dr. Jeff Henderson’s lab and were stored at 4 °C until processing (less than 24 h). Urine was filtered through a 40 μm mesh and centrifuged at 2000*g* for 12 min. Supernatant was immediately aliquoted into cryovials and stored at −80 °C until it was ready for experimental analysis (1 freeze/thaw cycle).

### S100A8/A9 Protein Quantification

S100A8/A9 levels were measured in all samples using the DuoSet ELISA Development Kit for human S100A8/A9 heterodimer (catalog no. DY8226, R&D Systems Inc.), according to the manufacturer’s instructions. Samples were diluted in reagent diluent (50 mM Tris, 10 mM CaCl_2_, 0.15 M NaCl, 0.05% Brij 35, pH 7.45–7.55, 0.2 μm filtered). Absorbance was read at 450 and 570 nm (for wavelength correction) using the Synergy HT Microplate ELISA reader (BioTek Inc.).

Sample dilutions for each cohort and biofluid were determined by serially diluting samples 2-fold to encompass ranges from 1:62.5 (initial 16 μL sample + 984 μL reagent diluent) to 1:4000. If results from the 1:62.5 dilution were below the range of detection, samples were diluted 1:25 and serially diluted to 1:200. The dilution factor was chosen based on raw calculated S100A8/A9 level (pg/mL) and absorbance within the detection range of the assay (94 to 6000 pg/mL). Urine samples were diluted 1:25, while all serum samples were diluted 1:4000.

### Statistics

A two-tailed Student’s *t* test analyzed the differences between the means of two groups. Multiple groups were analyzed via ANOVA using Tukey’s or Dunnett’s post-test, as indicated. Non-parametric alternatives were performed if the data were not normally distributed (Mann–Whitney U test instead of Student’s *t* test; Kruskal–Wallis test with multiple comparison analysis instead of an ANOVA). Simple correlation and linear regression analyses were performed with parametric (Pearson) and non-parametric analysis (Spearman) as indicated. A *p*-value of ≤0.05 was considered significant. Statistical analyses were performed in GraphPad Prism.

## Supporting Information Available

Patient demographics (Table S1); healthy cohort demographics (Table S2); ANOVA statistics table analyzing serum S100A8/A9 levels by length of patient hospitalization in relation to patient mortality, ICU admission, and ICU ventilation outcomes (Table S3); ANOVA statistics table analyzing serum S100A8/A9 levels by length of hospitalization in relation to patient BMI (Table S4); ANOVA statistics table analyzing serum S100A8/A9 levels by patient BMI in relation to patient mortality and ICU admission outcomes (Table S5); ANOVA statistics table analyzing urine S100A8/A9 levels by length of hospitalization in relation to patient mortality, ICU admission, and ICU ventilation outcomes (Table S6); COVID-19 serum and urine S100A8/A9 correlation analyses stratified by length of hospital admission (Figure S1) (PDF)

## Abbreviations

COVID-19: coronavirus disease 19
ICU: intensive care unit
BMI: body mass index
CRP: c-reactive protein
TPC: tissue procurement center
