# Supplementary material for: Elevated Level of Circulating but Not Urine S100A8/A9 Identifies Poor COVID-19 Outcomes
Source: ACS Infect Dis. 2023 Oct 3;9(10):1815–20. doi: 10.1021/acsinfecdis.3c00249 (PMC10580308; doi:10.1021/acsinfecdis.3c00249)
Supplement: Supplementary file 1 — id3c00249_si_001.pdf [file id3c00249_si_001.pdf]

Elevated levels of circulating but not urine S100A8/A9 identifies poor COVID-19 outcomes

Leah Mellett[a,b], Gaya Amarasinghe[b], Christopher W Farnsworth[b], Shabaana Khader[a,c,*]

[a]Department of Molecular Microbiology, Washington University in St. Louis, St. Louis, MO 63108, USA

[b]Department of Pathology and Immunology, Washington University School of Medicine, St Louis, MO 63108, USA

[c]Department of Microbiology, University of Chicago, Chicago, IL 60637 USA

[*]Email: khader@uchicago.edu

Table S1: COVID-19 Patient Demographics

Factor	N (serum)	N (matched serum + urine)
Total Patients	73	45
Age		
<60	16	6
60-70	22	15
71-80	22	15
>80	13	9
Sex		
Female	22	12
Male	51	33
Race		
Black	50	33
White	22	11
Asian	1	1
Mortality Outcome		
Alive	44	30
Deceased	29	15
ICU Admission		
Yes	49	33
No	24	12
ICU Ventilation Requirement		
Yes	26	14
No	23	19
BMI		
Underweight (<18.4)	4	2
Normal (18.5-24.9)	18	12
Overweight (25-29.9)	22	16
Obese (30-34.9)	13	8
Extremely Obese (35+)	16	7

Table S2: Healthy Patient Demographics (20 patients)

Factor	N
Age	
<20	1
20-30	5
31-40	10
41-50	4
Sex	
Female	11
Male	9
Race	
Black	7
White	13
BMI	
Underweight (<18.4)	0
Normal (18.5-24.9)	5
Overweight (25-29.9)	8
Obese (30-34.9)	3
Extremely Obese (35+)	4

Table S3. ANOVA Statistics Table for COVID-19 patient mortality, ICU, and Ventilation status v. length of hospitalization (serum)

	SS (Type III)	DF	MS	F (DFn, DFd)	p-value
Interaction	50481281	4	12620320	F (4, 209) = 0.2677	0.8985
Time	30066092	4	7516523	F (4, 209) = 0.1594	0.9585
Mortality	473812300	1	473812300	F (1, 209) = 10.05	0.0018
Residual	9852863069	209	47142885		
Interaction	137497655	4	34374414	F (4, 217) = 0.7115	0.5849
Time	250486829	4	62621707	F (4, 217) = 1.296	0.2725
ICU Admission	212903487	1	212903487	F (1, 217) = 4.407	0.0370
Residual	10484219329	217	48314375		
Interaction	136593095	4	34148274	F (4, 152) = 0.7153	0.5827
Time	67288169	4	16822042	F (4, 152) = 0.3524	0.8421
ICU Ventilation	165294821	1	165294821	F (1, 152) = 3.463	0.0647
Residual	7256220359	152	47738292		

Table S4. ANOVA Statistics Table for COVID-19 patient BMI v. length of hospitalization (serum)

	SS (Type III)	DF	MS	F (DFn, DFd)	p-value
Interaction	478754296	4	29922143	F (16, 202) = 0.6145	0.8703
Time	161466653	4	40366663	F (4, 202) = 0.8290	0.5081
BMI	518413425	1	129603356	F (4, 202) = 2.662	0.0338
Residual	9835459888	209	48690395		

Table S5. ANOVA Statistics Table for COVID-19 patient mortality and ICU v. BMI (serum)

	SS (Type III)	DF	MS	F (DFn, DFd)	p-value
Interaction	142964490	4	35741123	F (4, 54) = 0.6427	0.6344
BMI	240687280	4	60171820	F (4, 54) = 1.082	0.3746
Mortality	10121314	1	10121314	F (1, 54) = 0.1820	0.6714
Residual	3003140595	54	55613715		
Interaction	85978594	4	21494648	F (4, 54) = 0.3810	0.8213
BMI	236049978	4	59012494	F (4, 54) = 1.046	0.3922
ICU Admission	45566137	1	45566137	F (1, 54) = 0.8076	0.3728
Residual	3046634459	54	56419157		

Table S6. ANOVA Statistics Table for COVID-19 patient mortality, ICU, and Ventilation status v. length of hospitalization (urine)

	SS (Type III)	DF	MS	F (DFn, DFd)	p-value
Interaction	4041	3	1347	F (3, 70) = 0.6554	0.5822
Time	7472	3	2491	F (3, 70) = 1.212	0.3118
Mortality	3613	1	3613	F (1, 70) = 1.758	0.1892
Residual	143838	70	2055		
Interaction	10443	3	3481	F (3, 70) = 1.762	0.1623
Time	9025	3	3008	F (3, 70) = 1.523	0.2161
ICU Admission	5327	1	5327	F (1, 70) = 2.697	0.1050
Residual	138266	70	1975		
Interaction	5630	3	1877	F (3, 50) = 1.020	0.3916
Time	17889	3	5963	F (3, 50) = 3.242	0.0296
ICU Ventilation	326.2	1	326.2	F (1, 50) = 0.1774	0.6755
Residual	91975	50	1840		

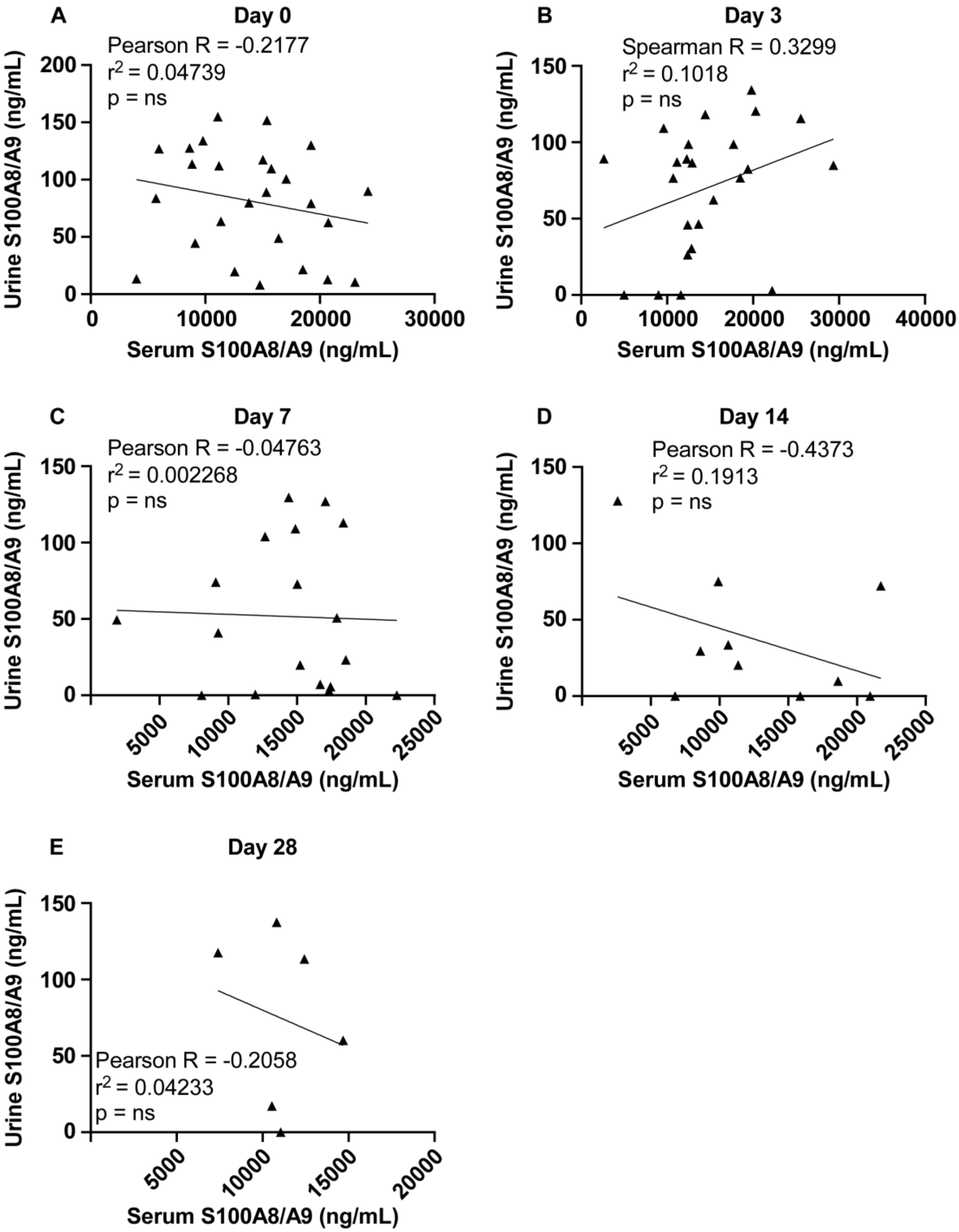

Figure S1. Correlation S100A8/A9 quantification analyses of matched urine and serum samples from COVID-19 patients stratified by day since hospital admission. Simple correlation and linear regression analysis.
